# Experiences and Predictors of Real‐Time Prescription Drug Monitoring Program Use Among Australian Community Pharmacists: A Nationwide Study

**DOI:** 10.1111/dar.70210

**Published:** 2026-07-10

**Authors:** Louisa Picco, Monica Jung, Rose Laing, Jarrod McMaugh, Hester Wilson, Suzanne Nielsen

**Affiliations:** ^1^ Monash Addiction Research Centre, Eastern Health Clinical School Monash University Melbourne Australia; ^2^ Pharmaceutical Society of Australia Melbourne Australia; ^3^ Centre for Alcohol and Other Drugs, NSW Ministry of Health Sydney Australia; ^4^ School of Population Health, Faculty of Medicine UNSW Sydney Sydney Australia

**Keywords:** community pharmacy, pharmacy practise, policy, prescription drug monitoring programs, real‐time prescription monitoring

## Abstract

**Introduction:**

Prescription drug monitoring programs (PDMP) are a key supply‐side policy aimed at reducing medication‐related harms, which have been implemented in all Australian states and territories; however, experiences have not been explored nationally among community pharmacists. The objectives of this study were to investigate PDMP use among Australian community pharmacists, including frequency of use, reasons for and barriers to use.

**Methods:**

An anonymous online survey collected data on: (i) the pharmacist (i.e., gender, years of pharmacy experience, PDMP training) and the pharmacy (i.e., location, pharmacy type, average number of daily prescriptions received); and (ii) PDMP use (i.e., frequency of use, reasons for checking PDMP, frequency of barriers experienced). Logistic regression was used to explore the factors associated with frequent PDMP use.

**Results:**

Among a sample of 730 community pharmacists, regular (‘all the time’) PDMP use was more common in mandated (74.1%), compared to non‐mandated (45.7%) states/territories (*p* < 0.001). Few pharmacists reported experiencing barriers ‘all the time’ in mandated (4.0%) and non‐mandated (4.8%) states/territories. Pharmacists working in mandated states/territories (aOR: 3.64; 95% CI 2.60–5.09), capital cities (aOR: 1.90; 95% CI 1.34–2.70) and pharmacies that are part of chain/banner group (aOR: 1.49; 95% CI 1.04–2.13) were more likely to use PDMP ‘all the time’, compared with those with less frequent use.

**Discussion and Conclusions:**

Australian community pharmacists demonstrate variations in the frequency and reasons for PDMP use. Concerted efforts through education and practise‐level interventions are needed to encourage PDMP use, particularly in non‐mandated states and territories, independent pharmacies, and those working outside capital cities.

## 1 Introduction

Drug‐related harms are a global public health concern, directly and indirectly impacting individuals, families, communities and society more broadly. Over the past two decades, the opioid crisis in North America has been marked by a shift from pharmaceutical to illicit opioid harm, driven in part by changing medicine access [1]. In contrast, Australia's drug‐related harms remain predominantly attributed to prescription medications such as opioids and benzodiazepines [2]. Medication‐related harms include fatal and non‐fatal overdoses, dependence, ambulance and hospital attendances, resulting in social, economic and health implications [3, 4]. In response, the World Health Organization's third Global Patient Safety Challenge, ‘Medication Without Harm’, aims to reduce medication related harms by 50% globally in the next 5 years [5].

Australia has implemented a range of multifaceted, multidisciplinary strategies, policies and interventions, including supply and demand side interventions, to reduce drug‐related harms [6]. An example of a nationally implemented supply‐side policy is Australia's real‐time prescription drug monitoring programs (PDMP). These electronic databases monitor the prescribing and dispensing of high‐risk medicines such as opioids, benzodiazepines, gabapentinoids, sedatives and stimulants [7]. The overall aims of these programs are to reduce nonmedical use of these high‐risk monitored medicines by identifying patients at risk of dependence or nonmedical use (through monitoring prescribed doses and high‐risk medicine combinations), reducing multiple prescriber episodes, detecting possible diversion, and supporting regulatory compliance [8].

Tasmania was the first Australian state to implement a PDMP in 2009 [9]. In 2018, the Australian government committed to national *real‐time* PDMP implementation, whereby all PDMPs capture prescribing and dispensing information at the time these processes occur. This national rollout commenced in the state of Victoria in 2019, with Western Australia being the last state to implement its real‐time PDMP in 2023. Australian PDMPs are commonly integrated into prescribing and dispensing software, delivering real‐time notifications and alerts. Community pharmacists use PDMPs to review patients' prescription and dispensing histories, enabling them to assess the clinical appropriateness of prescriptions, make informed decisions when dispensing, coordinate care with other clinicians involved in the patient's care, and intervene when there is potential for harm or misuse [8]. Despite their ability to inform clinical decision‐making, PDMP use has also resulted in unintended consequences, including abrupt medication and treatment refusal, increased stigma, substitution effects and people transitioning from prescription to illicit drug use [10, 11, 12, 13].

Several studies have explored Australian pharmacists' experiences of PDMPs in the states of Victoria, New South Wales, Queensland and Western Australia. These examined the frequency of use and how the PDMP informs clinical decision making, revealing distinct differences across these states [14, 15, 16]. To date, however, no study has explored PDMP experiences across all Australian states and territories. To address this gap, this is the first national Australian study to investigate PDMP use among community pharmacists and aims to explore frequency and reasons for use as well as barriers, comparing mandated and non‐mandated states and territories.

## 2 Methods

### 2.1 Design

Data were collected as part of a larger study on harm reduction provision and medicine safety best practises within community pharmacies [17], via an online anonymous survey, administered via Qualtrics. Utilising an established nationally representative sampling method [18], pharmacists were randomly approached via phone between January 2025 and April 2025, with the aim of recruiting at least a 10% sample of all pharmacies from each Australian state and territory. This study is reported in accordance with the STrengthening the Reporting of OBservational studies in Epidemiology (STROBE) Checklist (See Appendix A).

### 2.2 Setting

Australia's PDMPs are jurisdictionally bound and regulated, meaning individual states and territories are responsible for independently implementing their PDMP, resulting in differences in implementation timeframes, who can access them, mandates related to their use and the medications they monitor (See Table 1). Despite these operational differences, they all utilise a backend algorithm that triggers a traffic light notification and alert system to indicate medication‐related possible risk. Red notifications indicate high‐risk behaviours such as being on a high oral morphine equivalent daily dose (e.g., over 100 mg oral morphine equivalent daily dose), obtaining medications from multiple prescribers, or being prescribed high‐risk medication combinations. Amber notifications are less severe and typically include being on medium dose (e.g., between 50 mg and 100 mg oral morphine equivalent daily dose) or obtaining medications from multiple pharmacies, while green notifications indicate no apparent risk according to the PDMP algorithm.

**TABLE 1:** Australia's implementation of real‐time prescription drug monitoring programs (PDMP) and features by jurisdiction.

Jurisdiction	PDMP name	Implementation date	Mandated use	PDMP access
Australian Capital Territory	Canberra Script	February 2022	No	All health professionals who prescribe or dispense monitored medicines
New South Wales	SafeScript NSW	May 2022	No	Medical practitioners, nurse practitioners and pharmacists
Northern Territory	NTScript	March 2022	No	Prescribers (i.e., doctors, dentists, nurse practitioners, endorsed midwives and podiatrists) and pharmacists
Queensland	QScript	September 2021	Yes	Medical practitioners, pharmacists, nurse practitioners, endorsed midwives, dentists, podiatric surgeons and endorsed podiatrists
South Australia	ScriptCheckSA	April 2021	Yes	Doctors, pharmacists and other health professionals who prescribe drugs (e.g., dentists, nurse practitioners)
Tasmania	TasScript	May 2024	Yes	Prescribers of Schedule 8 medicines (i.e., doctors, dentists, nurse practitioners) and pharmacists
Victoria	SafeScript	April 2019	Yes	Doctors, nurse practitioners and pharmacists
Western Australia	ScriptCheckWA	March 2023	No	All health professionals who prescribe or dispense monitored medicines

### 2.3 Participants and Procedures

Pharmacies were identified via two publicly available marketing lists. These lists were merged, with duplicates removed. The overall list was then split into individual lists for each state and territory and were randomised within Excel using the ‘=rand()’ formula. Recruitment commenced at the top of each randomised jurisdiction‐level list, with the aim of enrolling at least 10% of pharmacies in each jurisdiction. Pharmacies were contacted by phone between January and April 2025, whereby the pharmacist‐in‐charge at the time of contact was invited to participate. In order to avoid intra‐site correlation and cluster bias, only one pharmacist‐in‐charge was invited to participate at each pharmacy. Where pharmacies could not be contacted on the first attempt, a further two attempts were made. Pharmacists who declined participation received no further contact. For pharmacists who agreed to participate, a survey link was sent to their preferred email, along with an online information sheet and consent form. To support participation, follow up emails were sent after 1 and 2 weeks from the initial invitation. Ethical approval for the study was granted by the Monash University Human Research Ethics Committee (No: 45196).

### 2.4 Measures

#### 2.4.1 Pharmacy and Pharmacist‐Related Questions

Pharmacists provided information about their pharmacy, including the location (i.e., state or territory), its geographical classification (capital city, other urban city, rural or remote), the pharmacy type (i.e., chain/banner group or a single independent pharmacy), the average daily prescription count and whether their pharmacy stocked naloxone and offered opioid agonist treatment. Pharmacists also provided data pertaining to their gender and years of experience as a pharmacist.

#### 2.4.2 PDMP‐Related Questions

A series of questions relating to PDMP use and experiences, similar to those used in earlier studies among the same population [14, 16], were included. Pharmacists were asked to indicate how frequently they used the PDMP and how frequently they experienced barriers to use in the past 3 months, with response options including: (i) all of the time; (ii) most of the time; (iii) sometimes; (iv) rarely; and (v) never. Pharmacists reported specific barriers they had experienced, where multiple responses could be selected: (i) PDMP being limited by not including handwritten scripts; (ii) time‐consuming log‐in process; (iii) lack of time; (iv) lack of knowledge or awareness of the PDMP; (v) lack of integration into dispensing software; and (vi) complexity of navigating the program. They were also asked to indicate the reasons for checking the PDMP, where multiple responses could be selected including: (i) anyone with a script for monitored medication; (ii) anyone who receives a red high‐risk notification; (iii) anyone who receives an amber notification; (iv) anyone on opioid agonist treatment; (v) when a new customer comes in with a script for a monitored medication; (vi) customer's appearance; (vii) customer's behaviour indicating suspected misuse; (viii) dose of script; and (iv) medication class. Pharmacists also indicated whether they had received PDMP training.

### 2.5 Covariates

The following covariates related to PDMP use were examined: (i) PDMP use requirement (mandatory vs. voluntary); (ii) pharmacy location (capital city vs. urban/rural/remote); (iii) pharmacy type (independent vs. chain); (iv) average daily script count ≤ 200 vs. > 200 scripts; (v) provision of opioid agonist treatment (yes or no); (vi) provision of naloxone (yes or no); (vii) years as pharmacist (≤ 15 vs. ≥ 15 years); and (viii) receipt of further education in PDMP (yes vs. no).

### 2.6 Statistical Analysis

Descriptive statistics were used to explore the frequency of PDMP use, reasons for checking the PDMP, frequency of barriers and whether pharmacists received PDMP training. *χ* ^2^ test was used to test these differences in mandated vs. non‐mandated states and territories. Logistic regression was used to examine the correlates of frequent PDMP use among pharmacists who used the PDMP ‘all the time’ compared with those who used the PDMP less frequently. Models were adjusted for the covariates listed above to account for potential confounding. Adjusted odds ratio (aOR) and 95% confidence intervals (CI) were reported to estimate the effect size and statistical significance. The model used listwise deletion, and participants who did not complete all questions (including later questions on PDMP related training) were not included in the primary regression model. To assess for any potential bias this may introduce, we conducted a sensitivity analysis without the variable ‘receipt of further education in PDMP’, which allowed for an increased sample size (from *n* = 530 to *n* = 686), enabling us to assess the robustness of the main findings. *p*‐value < 0.05 indicated statistical significance for all analyses. All data cleaning and analyses were conducted using StataCorp STATA, Version 18.5 BE [19].

## 3 Results

**FIGURE 1:**
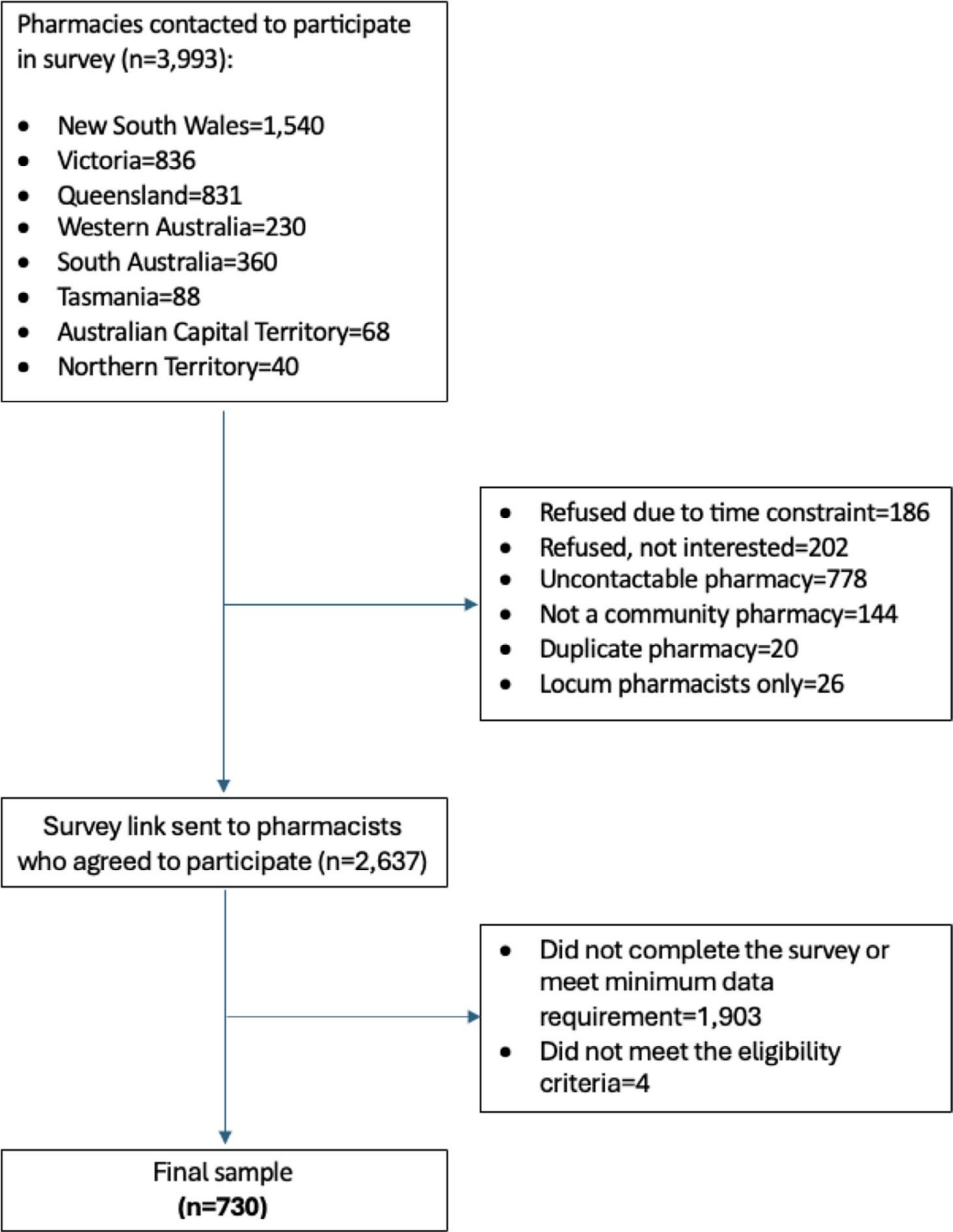
Sample recruitment.

The sample comprised 730 pharmacists, representing approximately 12% of Australian community pharmacies (See Figure 1). The majority of pharmacists were female (53.0%), with 38.5% having more than 15 years of pharmacy experience. Recruitment was representative of state and territory populations, resulting in the majority of pharmacists recruited from the three most populous states, New South Wales (31.4%), Victoria (23.8%) and Queensland (20.4%). One in four (40.1%) pharmacies were located in capital cities, while most (62.2%) pharmacies were part of a pharmacy chain or banner group (Table 2).

**TABLE 2:** Sample characteristics.

Pharmacist characteristics	n (%)
Gender^a^	
Male	340 (46.6)
Female	387 (53.0)
Age range, years	
< 25	24 (3.3)
25–34	259 (35.5)
35–44	224 (30.7)
45–54	137 (18.8)
55–64	65 (8.9)
≥ 65	21 (2.9)
Years of pharmacy practise	
≤ 5 years	180 (24.7)
6–10 years	135 (18.5)
11–15 years	134 (18.4)
16–20 years	94 (12.9)
> 20 years	187 (25.6)

Pharmacy characteristics	n (%)
Jurisdiction	
Australian Capital Territory	14 (1.9)
New South Wales	229 (31.4)
Northern Territory	8 (1.1)
Queensland	149 (20.4)
South Australia	59 (8.1)
Tasmania	20 (2.7)
Victoria	174 (23.8)
Western Australia	77 (10.6)
Pharmacy location	
Capital city	293 (40.1)
Urban/Rural/Remote	437 (59.9)
Pharmacy type	
Single independent	276 (37.8)
Chain/Banner group	454 (62.2)
Average daily script count	
≤ 200	399 (54.7)
> 200	331 (45.3)

^a^ < 0.5% reported they were non‐binary/gender diverse/prefer not to say.

Table 3 presents PDMP experiences among mandated and non‐mandated states and territories (See Appendix B for experiences by individual states/territories). Most pharmacists working in mandated states (i.e., Victoria, Queensland, South Australia and Tasmania) indicated that, in the past 3 months, they used their PDMP ‘all of the time’ (74.1%), while less than half of pharmacists (45.7%) in non‐mandated states (i.e., New South Wales, Western Australia, Australian Capital Territory and Northern Territory) used it ‘all of the time’. The most frequently reported reason for checking PDMP was ‘for anyone who receives a red high‐risk notification’ (mandated: 57.5%; non‐mandated: 60.7% *p* = 0.381). Significant differences were observed for some reasons for checking the PDMP between mandated and non‐mandated states, including: ‘for anyone with a script for monitored medication’ (mandated: 54.2%; non‐mandated: 35.1%; *p* < 0.001), ‘a customer's appearance’ (mandated: 26.9%; non‐mandated: 34.8%; *p* = 0.021), ‘when a customer's behaviour may indicate suspected misuse’ (mandated: 44.5%; non‐mandated: 55.8%; *p* = 0.002) and ‘the dose of the script’ (mandated: 45.3%; non‐mandated: 53.7%; *p* = 0.024) (Table 3).

**TABLE 3:** PDMP use and experiences for mandated and non‐mandated jurisdictions.

	Mandated PDMP use (n, %) *n* = 402 (55.1%)	Non‐mandated PDMP use (n, %) *n* = 328 (44.9%)	χ^2^	*p* value
Frequency of PDMP use				
All the time	298 (74.1)	150 (45.7)	72.1130	**< 0.001**
Most of the time	82 (20.4)	106 (32.3)		
Sometimes	18 (4.5)	54 (16.5)		
Rarely	3 (0.8)	13 (4.0)		
Never	1 (0.3)	5 (1.5)		
Reasons for checking PDMP^a^, ^b^				
Anyone with a script for monitored medication	218 (54.2)	115 (35.1)	26.7508	**< 0.001**
Anyone who receives a red high‐risk notification	231 (57.5)	199 (60.7)	0.7679	0.381
Anyone who receives an amber notification	183 (45.5)	130 (39.6)	2.5569	0.110
Anyone on opioid agonist treatment	97 (24.1)	60 (18.3)	3.6450	0.056
A new customer with a monitored medication script	209 (52.0)	185 (56.4)	1.4156	0.234
A customer's appearance	108 (26.9)	114 (34.8)	5.3138	**0.021**
A customer's behaviour indicates suspected misuse	179 (44.5)	183 (55.8)	9.1697	**0.002**
The dose of the script	182 (45.3)	176 (53.7)	5.0815	**0.024**
The medication class	170 (42.3)	127 (38.7)	0.9534	0.329
Frequency of experiencing barriers^c^				
All the time	15 (4.0)	14 (4.8)	11.0807	**0.026**
Most of the time	26 (7.0)	26 (8.8)		
Sometimes	169 (45.3)	162 (55.1)		
Rarely	149 (40.0)	82 (27.9)		
Never	14 (3.8)	10 (3.4)		
Received PDMP training^d^				
Yes	233 (77.7)	153 (66.5)	8.1720	**0.004**
No	67 (22.3)	77 (33.5)		

*Note:* Bold values indicate statistical significance (*p* < 0.05%).

Abbreviation: PDMP, prescription drug monitoring program.

^a^ Multiple response options could be selected.

^b^ Missing data *n* = 167.

^c^ Missing data *n* = 63.

^d^ Missing data *n* = 200.

The proportion of pharmacists experiencing barriers when using the PDMP was comparable among mandated and non‐mandated states and territories. Specifically, only a small proportion reported experiencing barriers ‘all the time’ (mandated: 4.0%; non‐mandated: 4.8%), while approximately half reported experiencing barriers ‘sometimes’ (mandated: 45.3%; non‐mandated: 55.1%). The most commonly reported PDMP‐related barriers included ‘PDMP being limited by not including handwritten scripts’ (75.8%), ‘time consuming log‐in process’ (62.3%), ‘lack of time’ (48.0%) and ‘lack of integration into dispensing software’ (23.6%). Significantly more pharmacists in mandated versus non‐mandated states and territories received PDMP training (mandated: 77.7%; non‐mandated: 66.5%; *p* = 0.004) (Table 3).

Table 4 presents the correlates of using the PDMP ‘all of the time’ compared with less frequent use. The logistic regression model revealed that pharmacists working in mandated states and territories (aOR: 3.20; 95% CI 2.18–4.71), capital cities (aOR: 1.74; 95% CI 1.16–2.62), pharmacies that are part of chain/banner group (aOR: 1.52; 95% CI 1.01–2.28), pharmacists with five or less years of pharmacy experience (aOR: 2.48; 95% CI 1.40–4.39) and pharmacists who received further education in PDMP (aOR: 2.04; 95% CI 1.32–3.16) were significantly more likely to use their PDMP ‘all the time’. Contrary to this, pharmacists who reported using their PDMP ‘all the time’ were significantly less likely to work in pharmacies that offer opioid agonist treatment (aOR: 0.60; 95% CI 0.40–0.89).

**TABLE 4:** Logistic regression showing the correlates of frequent PDMP use (*n* = 530).

	Use PDMP ‘all the time’ (n, %) *n* = 325 (61.3%)^a^	Less frequent use (n, %) *n* = 205 (38.7%)^b^	Adjusted odds ratio^c^ (95% CI)
PDMP implementation			
Mandatory	222 (74.0)	78 (26.0)	**3.20 (2.18–4.71)**
Voluntary	103 (44.8)	127 (55.2)	*Reference*
Pharmacy location			
Capital city	145 (69.7)	63 (30.3)	**1.74 (1.16–2.62)**
Urban, rural, remote	180 (55.9)	142 (44.1)	*Reference*
Pharmacy type			
Chain/banner group	212 (66.5)	107 (33.5)	**1.52 (1.01–2.28)**
Single independent	113 (53.6)	98 (46.5)	*Reference*
Average daily script count			
≤ 200	170 (59.0)	118 (41.0)	*Reference*
> 200	155 (64.1)	87 (36.0)	0.93 (0.62–1.39)
Offer opioid agonist treatment			
Yes	145 (56.0)	114 (44.0)	**0.60 (0.40–0.89)**
No	180 (66.4)	91 (33.6)	*Reference*
Stock naloxone			
Yes	247 (63.5)	142 (36.5)	1.38 (0.89–2.15)
No	78 (55.3)	63 (44.7)	*Reference*
Years as pharmacist			
≤ 5 years	88 (75.2)	29 (24.8)	**2.48 (1.40–4.39)**
6–10 years	58 (62.4)	35 (37.6)	1.49 (0.84–2.65)
11–15 years	54 (60.0)	36 (40.0)	1.26 (0.71–2.24)
16–20 years	44 (59.5)	30 (40.5)	1.47 (0.80–2.71)
> 20 years	81 (51.9)	75 (48.1)	*Reference*
Gender^d^			
Male	144 (57.6)	106 (42.4)	*Reference*
Female	180 (64.8)	98 (35.3)	1.41 (0.95–2.08)
Received further education in PDMP			
Yes	255 (66.1)	74 (51.4)	**2.04 (1.32–3.16)**
No	70 (48.6)	131 (33.9)	*Reference*

*Note:* Bold values indicate statistical significance (*p* < 0.05).

^a^ Missing data *n* = 123.

^b^ Missing data *n* = 77.

^c^ Adjusted for all covariates listed in this table.

^d^ ‘Other’ category for gender included non‐binary/gender diverse/prefer not to say (0.4%)—not reported in this table.

Abbreviations: CI, confidence interval; PDMP, prescription drug monitoring program.

A sensitivity analysis, which included the same covariates as the main analysis except ‘receipt of further education in PDMP’, showed comparable results with only minor, non‐significant differences in the odds ratios (See Appendix C). However, the sensitivity analysis revealed that pharmacists working in pharmacies that stocked naloxone were more likely to report using PDMP ‘all the time’ (aOR: 1.50; 95% CI 1.02–2.20), which was non‐significant in the main analysis.

## 4 Discussion

This is the first national Australian study among community pharmacists to explore PDMP use and experiences, following national real‐time prescription monitoring implementation. Results revealed that most Australian pharmacists use PDMPs regularly, with more frequent use among mandated states, consistent with an earlier study among a subset of four Australian states [16]. Although pharmacists reported barriers to PDMP use, these were infrequent but higher in non‐mandated states and territories. This finding may reflect less familiarity with the programs in these states and territories due to less frequent use and more recent implementation. Alternatively, this may reflect increased barriers in these states and territories, which may prohibit or deter more regular use.

Existing literature from the US has shown, unsurprisingly, that mandating PDMP use results in increased uptake and use; however, these effects are not always sustained [20, 21]. Furthermore, there is inconclusive evidence of the effects of such mandates. For example, PDMP mandates have been shown to reduce prescription opioid‐related morbidity and mortality and reduce behaviours synonymous with nonmedical use, including obtaining monitored medications from multiple prescribers [22, 23]. Contrary to this, others have demonstrated the effects of PDMP mandates on mortality rates, revealing decreasing prescription opioid‐related mortality but increasing heroin deaths, following the implementation of these mandates [13, 24]. With regards to the Australian context, early findings from Victoria show PDMP mandates did not impact high‐dose opioid prescribing or prescribing of high‐risk medication combinations [11], while opioid‐related emergency department presentation declined among people prescribed opioids [25], suggesting the implementation of this supply‐side policy has not had the same negative impacts observed in the US. The current study did however reveal that while some jurisdictions mandate PDMP use, pharmacists did not always use the PDMP as intended. This was most evident in South Australia and Tasmania, where just 58% and 65% of pharmacists, respectively, reported using it all of the time. Findings suggest there may be a need for ongoing education and socialisation of PDMPs among pharmacists to support their regular use and reinforce the benefits of these programs. Although checking the PDMP is not mandatory under some circumstances, these findings indicate that not all pharmacists are complying with their required obligations. This highlights the importance of ongoing evaluation of Australia's different PDMPs, in order to explore nuances and identify key features to support regular use, including under mandatory conditions, while monitoring for unintended consequences [26].

Significant differences in the reasons why pharmacists checked the PDMP were observed between mandated and non‐mandated states and territories. More pharmacists in mandated states and territories checked the PDMP ‘all the time’ for anyone with a monitored medication script, compared to those in non‐mandated states and territories, where checking the PDMP appeared to be a more targeted rather than a universal approach. While this finding is expected, given that pharmacists in mandated states are required to check the PDMP when a red or amber notification is triggered, it does suggest pharmacists may be relying heavily on this backend algorithm to determine when they check the PDMP, and not their own clinical judgement or other clinical indicators. PDMP algorithms are limited to identifying a small number of possible medication risks, and therefore, it is important that pharmacists continue to use their clinical expertise, alongside information within the PDMP [27]. When used consistently, checking the medication history in the PDMP may provide the opportunity to intervene before patients reach ‘risk’ thresholds. Interestingly, more pharmacists in non‐mandated states and territories used their PDMP based on a person's appearance and behaviour. This may reflect appropriate clinical judgement; however, it may also be an indication of underlying stigma, a well‐documented unintended consequence of PDMP implementation [10, 12]. Such stigma and discrimination can lead to unintended adverse consequences, such as treatment or medication refusal, which in turn can lead to untreated medical conditions or can drive patients to seek illicit substances [10, 28]. More recently, a range of educational resources and guidelines have been developed to support pharmacists in adopting a patient‐centred approach to identifying and mitigating possible risks [27, 29, 30], and may help to reduce stigma and avoid these unintended consequences.

Pharmacy and pharmacist‐specific characteristics were also associated with greater odds of pharmacists using the PDMP all of the time. PDMP mandates were the strongest predictor of regular PDMP use, with pharmacists working in mandated states and territories exhibiting over threefold greater odds of using the PDMP all of the time, compared with those in non‐mandated states and territories. Similarly, those who had received PDMP training had more than twice the odds of using the PDMP regularly. These are important findings and demonstrate the impact mandates and additional training can have on regular PDMP use. Pharmacists with fewer years of experience were also significantly more likely to report using the PDMP all of the time. These results are consistent with an earlier study among Victorian pharmacists, which also found more experienced pharmacists were less likely to use the PDMP, once use had been mandated [14]. Pharmacists located in capital cities were more likely to use PDMPs more frequently than those located outside of capital cities, which is consistent with an earlier finding among pharmacists in the Australian state of Victoria [14]. Those working within a pharmacy chain or banner group also had greater odds of using their PDMP all of the time. This may reflect the ability of pharmacy chains to leverage corporate infrastructure and implement centralised management structures, training systems, policies and procedures to support regular PDMP use [31, 32].

Pharmacists providing opioid agonist treatment were significantly less likely to use their PDMP all of the time. Although no data are available to explore this further, given that these medications are monitored by the PDMP, and that pharmacists have regular, often daily contact with these patients, it is possible that this patient familiarity and/or the burden of constantly checking the PDMP results in reduced PDMP use. This may also reflect alert fatigue [33], given that most people on opioid agonist treatment almost always will receive a red notification, and pharmacists may therefore undermine or ignore such notifications and alerts. This may also partially explain the less regular PDMP use for patients on opioid agonist treatment, with just 21% of pharmacists checking the PDMP all of the time. It is possible pharmacists feel they already have direct oversight over these patients, as opioid dependence medications are prescribed and dispensed under strict protocols and often have daily or supervised dosing [34]. This is unlikely to explain the overall trend for other patients, though; therefore, further exploration around the association between providing opioid agonist treatment and regular PDMP use is warranted.

### 4.1 Strengths and Limitations

This is the first national Australian study to explore PDMP experiences across all states and territories, particularly comparing mandated and non‐mandated states, utilising a robust representative sampling approach. The sample characteristics also resemble those from the Australian Health Practitioner Regulation Agency report [35], reflecting similarities to the overall pharmacy workforce, while the use of cognitively tested survey items from earlier studies strengthens the overall design. The following limitations should, however, be considered when interpreting the results. Recall and/or social desirability bias may have impacted some responses; however, measures such as making the survey anonymous would reduce these impacts. As the current sample includes one pharmacist‐in‐charge at each pharmacy, these findings relating to PDMP use and experiences may not be generalisable to all pharmacists. It is also possible that those more interested in the topic may be more likely to respond, which may lead to an overestimation of PDMP use in the sample. While pharmacists were required to report responses to all questions, not all pharmacists completed the entire survey, and the impact of missing data for some items cannot be fully explored.

## 5 Conclusions

Among a nationally representative sample of community pharmacists, key differences in the frequency of PDMP use and reasons for checking the PDMP were observed by state/territory and between mandated and non‐mandated states and territories. Specific characteristics such as mandates and receiving PDMP training were associated with more frequent use, while working outside of a capital city and the provision of opioid agonist treatment were associated with less frequent use. Concerted efforts through education and practise‐level interventions are needed to encourage PDMP use, particularly in non‐mandated states and territories, independent pharmacies and those working outside capital cities.

## Author Contributions

Conceptualization: LP, SN; Methodology: LP, MJ, RL, JM, HW, SN; Investigation: LP, MJ, RL; Formal analysis: MJ; Writing – original draft preparation: LP; Writing – review and editing: LP, MJ, RL, JM, HW, SN; Funding acquisition: LP, SN; Supervision: LP, MJ, SN.

## Funding

This work was supported by the National Health and Medical Research Council (2016909, 2025894).

## Conflicts of Interest

The authors declare no conflicts of interest.

## Data Availability

The data that support the findings of this study are available on request from the corresponding author. The data are not publicly available due to privacy or ethical restrictions.

## Appendix A See Table A1

**TABLE A1:** STROBE statement—checklist of items that should be included in reports of cross‐sectional studies.

	Item no	Recommendation	Page no
Title and abstract	1	(a) Indicate the study's design with a commonly used term in the title or the abstract	1
		(b) Provide in the abstract an informative and balanced summary of what was done and what was found	2
Introduction			
Background/rationale	2	Explain the scientific background and rationale for the investigation being reported	3
Objectives	3	State specific objectives, including any prespecified hypotheses	3–4
Methods			
Study design	4	Present key elements of study design early in the paper	4
Setting	5	Describe the setting, locations, and relevant dates, including periods of recruitment, exposure, follow‐up, and data collection	4
Participants	6	(*a*) Give the eligibility criteria, and the sources and methods of selection of participants	4–5
Variables	7	Clearly define all outcomes, exposures, predictors, potential confounders, and effect modifiers. Give diagnostic criteria, if applicable	5–6
Data sources/measurement	8^a^	For each variable of interest, give sources of data and details of methods of assessment (measurement). Describe comparability of assessment methods if there is more than one group	5–6
Bias	9	Describe any efforts to address potential sources of bias	—
Study size	10	Explain how the study size was arrived at	4
Quantitative variables	11	Explain how quantitative variables were handled in the analyses. If applicable, describe which groupings were chosen and why	5–6
Statistical methods	12	(a) Describe all statistical methods, including those used to control for confounding	6
		(b) Describe any methods used to examine subgroups and interactions	—
		(c) Explain how missing data were addressed	—
		(d) If applicable, describe analytical methods taking account of sampling strategy	—
		(e) Describe any sensitivity analyses	6
Results			
Participants	13^a^	(a) Report numbers of individuals at each stage of study—eg numbers potentially eligible, examined for eligibility, confirmed eligible, included in the study, completing follow‐up, and analysed	6
		(b) Give reasons for non‐participation at each stage	17
		(c) Consider use of a flow diagram	17
Descriptive data	14^a^	(a) Give characteristics of study participants (eg demographic, clinical, social) and information on exposures and potential confounders	6–7
		(b) Indicate number of participants with missing data for each variable of interest	—
Outcome data	15^a^	Report numbers of outcome events or summary measures	6–7
Main results	16	(*a*) Give unadjusted estimates and, if applicable, confounder‐adjusted estimates and their precision (e.g., 95% confidence interval). Make clear which confounders were adjusted for and why they were included	6–7
		(*b*) Report category boundaries when continuous variables were categorised	—
		(*c*) If relevant, consider translating estimates of relative risk into absolute risk for a meaningful time period	—
Other analyses	17	Report other analyses done—eg analyses of subgroups and interactions, and sensitivity analyses	20–22
Discussion			
Key results	18	Summarise key results with reference to study objectives	7–9
Limitations	19	Discuss limitations of the study, taking into account sources of potential bias or imprecision. Discuss both direction and magnitude of any potential bias	10
Interpretation	20	Give a cautious overall interpretation of results considering objectives, limitations, multiplicity of analyses, results from similar studies, and other relevant evidence	7–9
Generalisability	21	Discuss the generalisability (external validity) of the study results	10
Other information			
Funding	22	Give the source of funding and the role of the funders for the present study and, if applicable, for the original study on which the present article is based	10

*Note:* An Explanation and Elaboration article discusses each checklist item and gives methodological background and published examples of transparent reporting. The STROBE checklist is best used in conjunction with this article (freely available on the Web sites of PLoS Medicine at http://www.plosmedicine.org/, Annals of Internal Medicine at http://www.annals.org/, and Epidemiology at http://www.epidem.com/). Information on the STROBE Initiative is available at www.strobe‐statement.org.

^a^ Give information separately for exposed and unexposed groups.

## Appendix B See Table B1

**TABLE B1:** PDMP use and experiences by jurisdiction [*n*(%)].

	Mandated PDMP				Voluntary PDMP			Total *n* = 328 (44.9)
	VIC *N* = 174 (23.8%)	QLD *N* = 149 (20.4%)	SA *N* = 59 (8.1%)	TAS *N* = 20 (2.7%)	NSW *N* = 229 (31.4%)	WA *N* = 77 (10.6%)	ACT, NT *N* = 22 (3.0%)	
Frequency of PDMP use								
All the time	122 (70.1)	129 (86.6)	34 (57.6)	13 (65.0)	105 (45.9)	27 (35.1)	18 (81.8)	448 (61.4)
Most of the time	45 (25.9)	15 (10.1)	18 (30.5)	4 (20.0)	82 (35.8)	20 (26.0)	4 (18.2)	188 (25.8)
Sometimes	5 (2.9)	4 (2.7)	6 (10.2)	3 (15.0)	31 (13.5)	23 (29.9)	0	72 (9.9)
Rarely	2 (1.2)	0	1 (1.7)	0	8 (3.5)	5 (6.5)	0	16 (2.2)
Never	0	1 (0.7)	0	0	3 (1.3)	2 (2.6)	0	6 (0.8)
Reasons for checking PDMP^a^, ^b^								
Anyone with a script for monitored medication	83 (47.7)	97 (65.1)	27 (45.8)	11 (55.0)	85 (37.1)	16 (20.8)	14 (63.6)	333 (45.6)
Anyone who receives a red high‐risk notification	144 (65.5)	68 (45.6)	35 (59.3)	14 (70.0)	146 (63.8)	37 (48.1)	16 (72.7)	430 (58.9)
Anyone who receives an amber notification	96 (55.2)	53 (35.6)	21 (35.6)	13 (65.0)	95 (41.5)	25 (32.5)	10 (45.5)	313 (42.9)
Anyone on opioid agonist treatment	31 (17.8)	48 (32.2)	13 (22.0)	5 (25.0)	45 (19.7)	9 (11.7)	6 (27.3)	157 (21.5)
A new customer with a monitored medication script	92 (52.9)	71 (47.7)	32 (54.2)	14 (70.0)	136 (59.4)	36 (46.8)	13 (59.1)	394 (54.0)
A customer's appearance	56 (32.2)	29 (19.5)	21 (35.6)	2 (10.0)	85 (37.1)	21 (27.3)	8 (36.4)	222 (30.4)
A customer's behaviour indicates suspected misuse	90 (51.7)	51 (34.2)	28 (47.5)	10 (50.0)	136 (59.4)	35 (45.5)	12 (54.6)	362 (49.6)
The dose of the script	92 (52.9)	54 (36.2)	24 (40.7)	12 (60.0)	129 (56.3)	34 (44.2)	13 (59.1)	358 (49.0)
The medication class	68 (39.1)	66 (44.3)	25 (42.4)	11 (55.0)	95 (41.5)	22 (28.6)	10 (45.5)	297 (40.7)
Frequency of experiencing barriers^c^								
All the time	5 (3.1)	6 (4.4)	3 (5.3)	1 (5.6)	12 (5.9)	2 (2.9)	0	29 (4.4)
Most of the time	11 (6.8)	12 (8.9)	3 (5.3)	0	16 (7.8)	8 (11.6)	2 (10.0)	52 (7.8)
Sometimes	76 (46.6)	62 (45.9)	26 (45.6)	5 (27.8)	114 (55.6)	42 (60.9)	6 (30.0)	331 (49.6)
Rarely	65 (39.9)	51 (37.8)	22 (38.6)	11 (61.1)	55 (26.8)	16 (23.2)	11 (55.0)	231 (34.6)
Never	6 (3.7)	4 (3.0)	3 (5.3)	1 (5.6)	8 (3.9)	1 (1.5)	1 (5.0)	24 (3.6)
Received PDMP training^d^								
Yes	101 (74.8)	86 (81.1)	35 (81.4)	11 (68.8)	105 (65.2)	38 (66.7)	10 (83.3)	386 (72.8)
No	34 (25.2)	20 (18.9)	8 (18.6)	5 (31.3)	56 (34.8)	19 (33.3)	2 (16.7)	144 (27.2)

Abbreviations: ACT, Australian Capital Territory; NSW, New South Wales; PDMP, prescription drug monitoring program; QLD, Queensland; SA, South Australia; TAS, Tasmania; VIC, Victoria; WA, Western Australia.

^a^ Multiple response options could be selected.

^b^ Missing data *n* = 167.

^c^ Missing data *n* = 63.

^d^ Missing data *n* = 200.

## Appendix C See Table C1

**TABLE C1:** Logistic regression showing the correlates of frequent use of PDMP (*n* = 686).

	Use PDMP ‘all the time’ *N* = 418 (60.9%)^a^	Less frequent use *N* = 268 (39.1%)^b^	Adjusted odds ratio^c^ (95% CI)
PDMP implementation			
Mandatory	282 (74.8)	95 (25.2)	**3.66 (2.61–5.14)**
Voluntary	136 (44.0)	173 (66.0)	*Reference*
Pharmacy location			
Capital city	190 (69.1)	85 (30.9)	**1.93 (1.35–2.75)**
Urban, rural, remote	228 (55.5)	183 (44.5)	*Reference*
Pharmacy type			
Chain/banner group	284 (66.5)	143 (33.5)	**1.45 (1.01–2.09)**
Single independent	134 (51.7)	125 (48.3)	*Reference*
Average daily script count			
≤ 200	212 (56.7)	162 (43.3)	*Reference*
> 200	206 (66.0)	106 (34.0)	1.19 (0.84–1.71)
Offer OAT			
Yes	176 (55.5)	141 (44.5)	**0.67 (0.47–0.95)**
No	242 (65.6)	127 (34.4)	*Reference*
Stock naloxone			
Yes	318 (63.2)	185 (36.8)	**1.50 (1.02–2.20)**
No	100 (54.6)	83 (45.4)	*Reference*
Years as pharmacist			
≤ 5 years	119 (71.7)	47 (28.3)	**2.22 (1.37–3.61)**
6–10 years	76 (62.8)	45 (37.2)	1.56 (0.93–2.61)
11–15 years	77 (60.2)	51 (39.8)	1.37 (0.83–2.26)
16–20 years	53 (58.2)	38 (41.7)	1.38 (0.79–2.39)
> 20 years	93 (51.7)	87 (48.3)	*Reference*
Gender^d^			
Male	184 (58.2)	132 (41.8)	*Reference*
Female	232 (63.2)	135 (36.8)	1.22 (0.87–1.72)

*Note:* Bold values indicate statistical significance (*p* < 0.05).

Abbreviations: CI, confidence interval; OAT, opioid agonist treatment; PDMP, prescription drug monitoring program.

^a^ Missing data *n* = 30.

^b^ Missing data *n* = 14.

^c^ Adjusted for all covariates listed in this table.

^d^ ‘Other’ category for gender included non‐binary/gender diverse/prefer not to say (0.4%)—not reported in this table.
